# Child Sexual Abuse Victimization: Focus on Self-Compassion

**DOI:** 10.3389/fpsyt.2022.818774

**Published:** 2022-03-02

**Authors:** Christine Wekerle, Katherine Kim, Nikki Wong

**Affiliations:** ^1^Department of Pediatrics, McMaster University, Hamilton, ON, Canada; ^2^Temerty Faculty of Medicine, University of Toronto, Toronto, ON, Canada

**Keywords:** child welfare, self-compassion, child sexual abuse, child sexual exploitation, Indigenous

## Introduction

Child sexual abuse (CSA) refers to using a child for the sexual stimulation of an adult, involving contact (touching) or non-contact (not touching) interactions. Where legal age of majority is required for consent, all sexual acts between an adult and underage children, by definition, are CSA. Most U.S. states require investigation into CSA, rather than an alternate child welfare service response (1). In North America, the prevalence of CSA is 20.4% for girls and 14.1% for boys, though it is likely much higher than reported due to low rates of disclosure (2). In comparing countries, there is a consensus in terms of contact behaviors, such as fondling and rape (3). Non-contact CSA (i.e., exposure to pornography, verbal sexual harassment, taking sexual photos) is less clearly included in definitions. However, research has shown that non-contact CSA is linked with adolescent distress and poorer quality of life, as compared to no-CSA youth (4). With over 10 years of medical data tracking, substantiated CSA youth were over three times more likely to present with a mental health disorder (e.g., depression, post-traumatic stress disorder, anxiety, behavioral problems, etc.), as compared to the general population. Over 20% of CSA youth had 2 to 3 diagnoses, and over 22% had 4+ diagnoses (5). CSA and psychiatric diagnoses were found to be stronger with self-reported childhood sexual abuse (CSA) than with agency-established CSA (6). While the personal strain to the CSA victim is inestimable, in terms of services and associated costs, the lifetime burden of CSA in the U.S. is placed at $9.3 billion (7).

Technically, child sexual exploitation (CSE) is CSA (8). CSE is defined as the involvement of a minor in sexual activity in exchange of something (e.g., money, food, shelter, etc.) from a perpetrator or third-party (e.g., recruiter, trafficker, etc.) (9). Most sex trafficking victims (88.5% girls) are between 14 and 17 years old. Up to a third of these sex trafficking victims also had reports of CSA and neglect (10). It is important to consider, not only all sexual abuse-related forms (CSE), but also all types of maltreatment (11, 13).

Vulnerable youth groups (e.g. gender-diverse, youth of color, Indigenous, refugees) experience systemic CSA risk factors. Youth with histories of greater poly-victimization are more likely to experience maltreatment while in foster care, compared to those with fewer maltreatment experiences (12). CSA in a child welfare system-involved adolescent sample seemed under-detected by caseworkers when considering youth self-report, particularly for boys (13). According to the Canadian Census 2016 data, Indigenous children accounted for 52.2% of children in foster care, despite only comprising 7.7% of the child population in Canada, highlighting potential organizational betrayal trauma with multiple care placements (14, 15). In a sample of American Indigenous adults with Type 2 diabetes, 29.1% reported experiencing CSA (16). Youth with CSA backgrounds “cross-over” systems, such as from child welfare to juvenile justice, which may challenge continuity in trauma-informed care (17). Black adolescent girls experience higher rates of sexual violence than girls of other races [11.2% forced intercourse, (18)], factoring into the CSA to CSE “pipeline” in juvenile justice (19, 20). There is evidence for the under-reporting of male CSE victims (21), see ECPAT's Global Boys Initiative (22).

The on-line environment has also been a context for higher CSA risks. Nagata et al. (23) reported youth (aged 12 to 13 years) spend an average of 7.7 h daily online for entertainment, notably on streaming and gaming platforms. In tracking pandemic impacts, Canada reported a 37% increase in child luring *via* a computer, as well as an up to 27% increase in the production and distribution of child sexual abuse material (CSAM) (24). It is important to appreciate that CSAM represents re-victimization, with image take-downs being difficult to maintain on the surface and dark web. Research shows that CSAM is readily located, with billions of images in circulation (25). In terms of clinical impact, the distinctions among types of CSA are less important than considering these as different aspects of CSA. In this opinion article, we advance that CSA-related trauma and on-going impairment stems from self-dysfunction, and that self-compassion as a clinical target is a critical component to trauma-informed care.

## CSA, Post-Traumatic Stress and the Self

Traumatization challenges the development of the self toward a competent identity (26, 27). The self-trauma model (28) identifies that early and repeated CSA may disrupt self-schemas and attachment models, as verbal mediation strategies (e.g., self-talk, self-expression) are less available when there is strong classical conditioning (e.g., abuse actions eliciting fear). If there are multiple perpetrators, the one constant is the presence of self; the self and self-emotions (e.g., shame) may come to function as threatening, requiring some perceptual avoidance (29). CSA has been strongly associated with a range of psychological and physical problems in childhood and adulthood, particularly post-traumatic stress disorder (PTSD) (30). PTSD symptomatology specifies three key areas: re-experiencing, avoidance and arousal. Although not without criticism (31), the DSM-5 added two new criteria as part of the new symptom cluster D of negative cognitions and mood: “Persistent, distorted cognitions about the cause or consequences of the traumatic event(s) that lead the individual to blame himself/herself or others” (p. 4), as well as “persistent negative emotional state (e.g., fear, horror, anger, guilt, or shame)” (p. 3). In trauma-exposed foster children, aged 5 to 8, PTSD diagnosis rates were 54% of children (32). Among juvenile detainees, CSA predicted PTSD symptoms, over and above other forms of trauma (17), suggesting unique impact of CSA. Lack of safety is salient within an attachment framework for PTSD, with insecure attachment linked with greater PTSD diagnosis and symptoms among children with CSA experiences, compared to those without (33, 34). Disrupted mentalizing may underlie self-conceptualization. Mentalization refers to the ability to reflect and understand what one is feeling, as well as the ability to understand others' affective states, which is a process of imagination (35, 36). The mentalizing function, with a secure attachment model, would enhance the development of openness, acceptance, curiosity, playfulness, perspective-taking, and self-compassion. A mentalizing deficit may be a form of inhibition or phobic reaction to mentalizing. In CSA, mentalizing about the self may lag in terms of learning self-compassion from the caregiving environment, with fewer opportunities to practice applying re-appraisal cognitions (positive reframing), or use constructive emotion (loving-kindness) to counteract distress (feeling bad about self) (33). The view of the self may be directly impacted by repeated perpetrator communications (e.g., telling the child “you are nothing”; “you are a slut”; “you want this” etc.), at ages when self-identity is more emergent (under age 8) or more solidified (adolescence to young adulthood). Both are peak ages for CSA (37). Defensive mentalizing (e.g., detachment from emotions), helpful to the survivor in the victimizing environment, may generalize to all emotions, such that positive emotions about the self are more difficult to engage with. Defensive mentalizing could also include restricted emotionality, as with a predominance of fear and anxiety. With post-traumatic stress, triggers for high arousal may trigger anti-mentalizing. As such, self-compassion may be especially challenging to acquire and enact, where a fear of self-compassion may be present.

## CSA and Suicidality

Stigma and betrayal have been recognized as psychological burdens put upon victims of CSA (38). Adaptation responses may include the development of alexithymia (difficulty in identifying and describing feelings), which has been shown to impact the CSA—suicidality relationship (39). A self-punitiveness model (40) posits that sensitization to negative self-worth leads to an over-generalization of the self as inadequate, preferencing confirmatory experiences, including self-harm. This may serve a short-term function—rigid adherence to being overly self-reliant, maintaining high standards—but a longer-term dysfunction (41). Persistent trauma may be a factor in self-injury and suicidality, in part, related to PTSD symptoms (42) and a trait-level, persistent negative self-concept (40). The most final outcome is the victim's early mortality by suicide (7).

Angelakis et al. (43) and Angelakis et al. (44) found that for suicide attempts (to age 24), the highest rate was for CSA (*OR* = 3.41), and CSA was associated with a 4-fold increase in having made plans for suicide. In Liu et al.'s (45) review, a medium effect size was found for the relationship of CSA to non-suicidal self-injury. CSA was associated with an over 6-fold increase in repeated self-harm, with CSA being linked with loss and shame (46). Violent victimization is predictive of Indigenous boys' and girls' suicidal ideation and attempts (47), although CSA was not uniquely measured. Increased suicidality among Indigenous youth may reflect, in part, self-colonization with the Indigenous genocidal legacy (48). CSA is directly linked longitudinally to non-suicidal self-harm and suicide attempts among justice system-involved girls (49), and to suicide attempts among adult prisoners [58% male; (50)]. Early age of CSA onset (before age 9) is linked with increased suicidal intent, particularly with co-occurrence with physical abuse, predicting age at first suicide attempt (51). In a meta-analytic review of studies, the population attributable risks for the female and male CSA survivors was found to be about 22% (females) and 11% (males) for suicidal behavior (52). In a review of only longitudinal studies of suicidality in youth and young adults, the population attributable risk level was 14.3% (53). In a large study of child advocacy clinics, 1 in 3 youth reported that they “sometimes want to die” (54). While it has been argued that development is guided by innate self-righting (55), for the CSA youth survivor, maintaining a positive affiliation to the self is a very real issue. PTSD-related anger mediates the CSA—adolescent problem drinking relationship in child welfare-involved youth (56). Anger may not be evident until social comparison and conceptualizations increase in adolescence (57, 58). Self-harm and suicidality may be, in part, the failure of the meta-cognitive, reflecting functioning, where the adolescent self is understood as having capacity for protective anger and self-care (59). Supporting self-compassion may re-build mentalizing toward loving-kindness.

## Supporting Self-Compassion and Dampening Fear of Self-Compassion

Self-compassion is a malleable protective factor for trauma exposed youth (60). It may be an important therapeutic target for CSA victims with PTSD symptoms (61, 62). Self-compassion is derived from balance among the emotion regulation systems of threat (sympathetic nervous system) and soothing (parasympathetic nervous system), with various factors impacting motivation to act (63). Self-compassion is conceptualized by Neff and McGehee (64) as reflecting three distinct components: (1) self-kindness (i.e., kindness to oneself in adversity or failure), (2) common humanity (i.e., recognition that one's personal experiences are part of a greater human experience), and (3) mindfulness (i.e., balance of negative thoughts and emotions with awareness and presence). Fear of self-compassion relates to feelings that self-compassion is undeserved, unfamiliar, harmful or unwanted (65, 66). Boykin et al. (67) found that females reporting moderate to severe childhood maltreatment endorsed significantly higher levels of fear of self-compassion, as compared with participants with minimal to no childhood maltreatment. Self-compassion may be an important resilience target to address chronic self-issues (i.e., shame), as well as acute distress (i.e., self-hate) (68). Self-compassion may support CSA youth in various problems related to the self, such as body shame, self-efficacy, and self-concept (69–71). Generally, self-compassion has been positively related to body appreciation, such as protecting women's body appreciation from social comparisons and appearance contingent self-worth (72). In a meta-analytic review of adolescent self-compassion studies, a large effect size was noted in its impact on reduced distress [i.e., *r* = −0.55; 95% CI −0.61 to −0.47; (73)]. With regards to child welfare-involved youth, higher self-compassion was linked with lower depression, problem alcohol use, and suicidality substance (74). Self-compassion has also been shown to have a protective effect against alcohol-related problems in First Nations youth, with Spillane et al. (75) demonstrating a significant positive association between alcohol use and alcohol-related problems among First Nations youth with low self-compassion levels. However, Miron et al. (76) found a significant indirect effect of CSA on PTSD *via* fear of self-compassion, rather than self-compassion. Self-compassion may counter an overactive threat system and avoidant responses to high arousal. Adopting a self-compassionate stance is associated with less shame (77, 78) and decreased self-criticism (79, 80). For adolescents who, developmentally, are highly engaged with socially-prescribed self-identity, social self-compassion (e.g., managing social blunders, failing to meet social expectations) may be especially relevant (41).

Self-compassion resonates in various Indigenous cultures. For example, Haudenosaunee perspectives include kindness and gratitude as part of the concept of “good mind, ” which is more communally defined in terms of the power of the mind that is unified amongst relatives than individually-focused (81). The Mohawk word for compassion translates as “love among us” (A. General, personal communication, November 16, 2021). The Haudenosaunee perspective of self-compassion is self-love as a gifted creation. The Great Law of Peace directs balancing the principles of compassion for relations and the strength of self-love. In one sample of American Indigenous people, self-compassion enhanced one's feeling of belongingness, thus allowing for a better connection with community (82). Self-compassion was also associated with a decreased suicide risk in American Indigenous-identifying individuals (82).

Recent factor analytic work suggests refinement on how to view self-compassion components. In testing the factor structure of self-compassion, Strickland et al. (83) showed that, in a collegiate sample, a total score was not indicated. Instead, a two-factor higher-order model, with six lower-order factors best described the data. These two factors were labeled as self-caring and self-coldness. The lower-order factors for self-caring were: self-kindness, mindfulness, and common humanity. Lower-order factors for self-coldness were: self-judgment, over-identification, and isolation. Presumably, activating self-caring would be protective for managing self-coldness; self-coldness would be an intervention target to address punitiveness. The fear of self-compassion seems to represent two factors, one related to internalized social standards (i.e., flaws, failings), and one related to “emergency” emotionality (i.e., vulnerability, grief) (84). CSA may alter the view of the “self-in-the-world” as highly vulnerable, where self-compassion is not warranted or very contradictory to the self-schema. Fear of self and fear of self-compassion may relate to the lack of a protective presence in the overall experience of the CSA victim. Factor analytic studies on both self-compassion and fear of self-compassion scales show a commonality of the self-fears.

In reviewing self-compassion, PTSD and trauma, Winders et al. (85) included 35 studies where the mean age was 18 years old, with 11 of these being self-compassion intervention studies. Most self-compassion intervention studies showed improvements in PTSD symptoms and trauma-related guilt and shame; however, adolescents with CSA histories were not specifically considered in comparison to non-CSA youth. CSA interventions may include an emotion-focused therapy approach where protective anger helps to motivate self-protective, adaptive action (86). A meta-analysis of self-compassion interventions revealed significant improvement compared to controls in the following outcomes: self-compassion, stress, depression, mindfulness, self-criticism, and anxiety. Among these, depression symptoms improved and self-compassion was maintained over follow-up (87). While the differing impacts of intervention types were not analyzed, the types of interventions included both group and individual approaches referring to self-compassion theory. Recently, this approach has been combined with self-compassion training in an on-line program delivery model (88). Halamová et al. (88)'s on-line program, Emotion Focused Training for Self-Compassion and Self-Protection found improvements in both self-compassion and self-hatred among a general community sample, recruited through social media sites and health forums (control group mean age of 25.35 years; intervention group mean age of 33.73 years). Self-compassion has also been implemented in the mental health app, JoyPopTM, which includes a journal feature with writing prompts on the topic of self-compassion (e.g., “You should talk to yourself like someone you love. What are some qualities that you love about yourself?”). The app as a whole encourages a positive and kind view of the self, which can be replicated in other interventions (89). Interventions designed to develop a resilient self that address both the bolstering of self-compassion and the reduction of fear of self-compassion may be meritorious for those with CSA experiences, especially youth with intersectional traumas.

## Conclusion

There is a clear global consensus for the illegality and wrong of CSA, how it is an infringement of child rights in the highest order, and how CSA, CSE, and CSAM prevention is a high value, coordinated target for public health. The increasing trends in CSAM is a warning call to invest strongly in prevention, early intervention, and developmentally timed intervention. Adolescence is a key timeframe for self-identity and is a time of high on-line engagement; it is critical for youth to grow in emerging adulthood in safe, supportive environments, and practice adaptive emotion regulation skills. A population study of youth with and without substantiated CSA showed that CSA and substance misuse are significantly associated with the age at onset of psychotic disorders (90). Traub and Boynton-Jarrett (91) specify modifiable factors to bolster resilience, including fostering positive appraisal styles and self-care skills. We would add self-compassion to this listing of therapeutic strategies for effortful regulation of the self and consider self-compassion interventions (tackling also fear of self-compassion) as a good-fit model for addressing CSA-related impact. Anticipatory guidance and our duty-to-care propels greater empirically-based innovations that consider self-compassion in supporting CSA victims, from detection and disclosure to post-traumatic growth. It seems imperative that full measurement of self-compassion, including fear of self-compassion, is built into measurement models for intervention research, and that diverse populations, including those at higher risk of CSA, are included in studies. Studies on fear of self-compassion interventions are notably limited and a research gap to be filled. Coherent work is needed to evaluate its potential good fit for youth with CSA experiences. CSA is an experience put upon youth who are protected from this in law, yet whose very conceptualization of themselves and their feelings is jeopardized and whose capacity to survive and thrive is undermined. Preventing suicidality directs toward prevention of CSA, CSE and CSAM. Trauma-informed care prioritizes client psychological safety and trauma research needs to invest in understanding the potential of self-compassion interventions for our young people.

## Author Contributions

CW contributed to the majority of the writing and research. KK and NW supported in the writing and research. All authors edited and revised. All authors contributed to the article and approved the submitted version.

## Funding

This work was funded by the Canadian Institutes of Health Research Indigenous Gender and Wellness Grant number IWD-171382.

## Conflict of Interest

The authors declare that the research was conducted in the absence of any commercial or financial relationships that could be construed as a potential conflict of interest.

## Publisher's Note

All claims expressed in this article are solely those of the authors and do not necessarily represent those of their affiliated organizations, or those of the publisher, the editors and the reviewers. Any product that may be evaluated in this article, or claim that may be made by its manufacturer, is not guaranteed or endorsed by the publisher.
